# Thromboembolic Events Associated with Thalidomide and Multimodality Therapy for Soft Tissue Sarcomas: Results of RTOG 0330

**DOI:** 10.1155/2012/659485

**Published:** 2012-04-24

**Authors:** J. M. Kane, J. Harris, W. G. Kraybill, D. C. Harmon, D. S. Ettinger, D. R. Lucas, T. F. DeLaney, D. Wang, W. J. Curran, B. L. Eisenberg

**Affiliations:** ^1^Department of Surgical Oncology, Roswell Park Cancer Institute, Buffalo, NY 14263, USA; ^2^RTOG Statistical Center, Philadelphia, PA 19103, USA; ^3^Department of Surgical Oncology, The Ohio State University, Columbus, OH 43210, USA; ^4^Department of Medical Oncology, Massachusetts General Hospital, Boston, MA 02114, USA; ^5^Department of Medical Oncology, Johns Hopkins University, Baltimore, MD 21202, USA; ^6^Department of Pathology, University of Michigan, Ann Arbor, MI 48109, USA; ^7^Department of Radiation Oncology, Massachusetts General Hospital, Boston, MA 02114, USA; ^8^Department of Radiation Oncology, Medical College of Wisconsin, Milwaukee, WI 53226, USA; ^9^Department of Radiation Oncology, Winship Cancer Institute, Atlanta, GA 30322, USA; ^10^Department of Surgical Oncology, Norris Cotton Cancer Center, Lebanon, NH 03756, USA

## Abstract

*Introduction*. RTOG 0330 was developed to address the toxicity of RTOG 9514 and to add thalidomide (THAL) to MAID chemoradiation for intermediate/high grade soft tissue sarcomas (STSs) and to preoperative radiation (XRT) for low-grade STS. *Methods*. Primary/locally recurrent extremity/trunk STS: ≥8 cm, intermediate/high grade (cohort A): >5 cm, low grade (cohort B). Cohort A: 3 cycles of neoadjuvant MAID, 2 cycles of interdigitated THAL (200 mg/day)/concurrent 22 Gy XRT, resection, 12 months of adjuvant THAL. Cohort B: neoadjuvant THAL/concurrent 50 Gy XRT, resection, 6 months of adjuvant THAL. Planned accrual 44 patients. *Results*. 22 primary STS patients (cohort A/B 15/7). Cohort A/B: median age of 49/47 years; median tumor size 12.8/10 cm. 100% preoperative THAL/XRT and surgical resection. Three cycles of MAID were delivered in 93% cohort A. Positive margins: 27% cohort A/29% cohort B. Adjuvant THAL: 60% cohort A/57% cohort B. Grade 3/4 venous thromboembolic (VTE) events: 40% cohort A (1 catheter thrombus and 5 DVT or PE) versus 0% cohort B. RTOG 0330 closed early due to cohort A VTE risk and cohort B poor accrual. *Conclusion*. Neoadjuvant MAID with THAL/XRT was associated with increased VTE events not seen with THAL/XRT alone or in RTOG 9514 with neoadjuvant MAID/XRT.

## 1. Introduction

In 1995, the Radiation Therapy Oncology Group (RTOG) initiated 9514, a phase II study of neoadjuvant MAID (Mesna, Adriamycin, Ifosfamide, Dacarbazine) chemoradiation for high-risk STS of the extremity and body wall. Patients with large (≥8 cm), high-grade STS were scheduled to receive 3 cycles of neoadjuvant MAID chemotherapy with interdigitated preoperative radiation therapy (44 Gy given in two 22 Gy courses) followed by surgical resection and then 3 additional cycles of adjuvant MAID chemotherapy. The results for 64 evaluable patients were published in 2006 [1]. With a median tumor size of 15 cm, the overall limb salvage rate was 91% and 23% had a complete pathologic response following surgical resection. In addition, the estimated 5-year overall survival was a promising 71.2%. However, this multimodality regimen was not without significant treatment-related toxicity. Grade 4 toxicity occurred in 81% of patients, 13% experienced significant wound complications, and there were 3 treatment-related deaths.

Based upon the experience with 9514 and discussions with Clinical Trials Evaluation Program (CTEP) at the NCI, the Sarcoma Working Group within the RTOG developed protocol 0330, which attempted to minimize the toxicity seen with 9514, included low-grade tumors, incorporated biologic endpoints, and added thalidomide to the systemic chemotherapy regimen. As a “nontraditional” systemic therapeutic agent, thalidomide was chosen for several reasons. It has both antiangiogenic and immunomodulatory properties. Thalidomide is also a well-tolerated oral agent frequently used in combination therapy for other cancers. Finally, there appears to be a potential relationship between circulating levels of antiangiogenic regulators (bFGF, VEGF,and endostatin) and STS with a potential correlation between high VEGF levels and impaired survival [2, 3]. The purpose of this paper is to report on the available results for 0330, particularly the observed thromboembolic complications from the use of thalidomide with multimodality therapy.

## 2. Materials and Methods

### 2.1. Patient Eligibility

Eligible patients were ≥16 years old with a primary T2a or T2b STS (AJCC, 6th ed.) located on the upper extremity (including the shoulder), the lower extremity (including the hip), or the trunk. Patients with locally recurrent STS were also eligible provided that there was no evidence of metastatic disease and there had been no prior primary tumor site radiation. Patients were divided into cohort A and cohort B based upon the histologic grade of the sarcoma. Cohort A consisted of patients with grade 3 or 4 (intermediate to high grade) STS ≥ 8 cm in maximal diameter. Cohort B included patients with grade 1 or 2 (low grade) STS > 5 cm in size. Given the planned adriamycin in cohort A, patients also had to have acceptable heart function. Due to the known thromboembolic risks and side effects associated with thalidomide, exclusion criteria for both cohorts included a known history of deep vein thrombosis or pulmonary embolus (except in patients where the cause was directly related to foreign body implants, i.e, a central venous catheter), a known hypercoagulable disorder, baseline CTCAE v3.0 grade 2 or greater fatigue or other global neurocognitive symptomatology, taking sedating drugs, not agreeing to avoid sedating illegal “recreational” drugs or alcohol greater than one drink per day, or known acquired immune deficiency syndrome. Finally, given the teratogenic effects, pregnancy was an absolute contraindication and both male and female patients agreed to follow all contraceptive requirements.

### 2.2. Radiation Therapy

Preoperative radiation therapy for cohort A (patients receiving MAID chemotherapy) consisted of two courses of external beam radiation therapy (EBRT) interdigitated between each course of MAID. Each course of EBRT was to begin 3 days after completion of each cycle of MAID and consisted of 22 Gy in 11 fractions (once a day) over 15 days. The total planned preoperative radiation dose was 44 Gy in 22 fractions. Thalidomide was to be given 7 days per week each evening before bedtime during the radiation phase of the therapy but was not to be given concurrent with the chemotherapy. Preoperative radiation therapy for cohort B included 50 Gy in 25 fractions, delivered at 2 Gy per fraction daily over 5 weeks. Thalidomide was to be given 7 days per week each evening before bedtime during the radiation phase of therapy.

A postoperative EBRT boost was given for patients with a positive margin. This consisted of 16 Gy in 8 daily fractions to the bed of the residual tumor (including a margin of 1 cm). A boost was not given for patients with 100% necrosis. Postoperative EBRT began approximately 2 weeks following resection, assuming there was satisfactory healing of the surgical wound.

Conventional radiotherapy simulated with a 2D simulator and conformal radiotherapy planned with a CT simulator were acceptable treatment techniques, but intensity modulated radiation therapy (IMRT) and brachytherapy were not permitted.

### 2.3. Chemotherapy and Thalidomide

**Figure 1:**
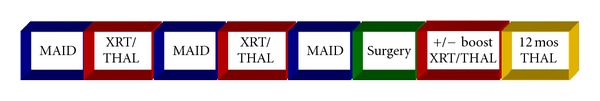
Treatment schema for RTOG 0330 cohort A. Neoadjuvant MAID (Mesna, Doxorubicin, Ifosfamide, DTIC) × 3 cycles, concurrent thalidomide (THAL) and radiation therapy (XRT) × 2 cycles, followed by surgical resection, followed by adjuvant THAL for one year (post-op boost XRT if a positive margin).

Patients in Cohort A were scheduled to receive a maximum of 3 cycles of neoadjuvant MAID with concurrent thalidomide/radiation therapy. This was followed by surgical resection and then adjuvant thalidomide for one year. The treatment schema is summarized in Figure 1. The study utilized the Common Terminology Criteria for Adverse Events (CTCAE) version 3.0 for grading of all treatment related adverse events. The 3 cycles of MAID consisted of the following. 

*Mesna*: 2500 mg/m^2^/day as a continuous infusion for 4 days starting on day 1 and repeated on day 22 and day 43 for 4 days. 

*Doxorubicin*: 20 mg/m^2^/day as a continuous infusion for 3 days starting on day 1 repeated on day 22 and day 43 for 3 days.

*Ifosfamide*: 2500 mg/m^2^/day as a continuous infusion for 3 days starting on day 1 and repeated on day 22 and day 43 for 3 days.

*DTIC*: 225 mg/m^2^/day as a continuous infusion for 3 days starting on day 1 and repeated on day 22 and day 43 for 3 days.

*G-CSF*: 5 mcg/kg/day subcutaneously starting on day 5 (24 hours after completion of chemotherapy) and continuing daily until the WBC count recovered.

At the beginning of the preoperative radiation phase, thalidomide was started at 200 mg/day. Thalidomide was not administered during the MAID chemotherapy. Two weeks after surgery or when the postoperative radiation began (for the subset that needed a boost for positive margins), thalidomide was restarted at 200 mg/day (or at the last well-tolerated dose). The postoperative thalidomide was administered daily for one year (regardless of the boost radiation schedule). If patients did not develop neuropathy at the 200 mg/day dose, the treating physician had the option to dose-escalate to 400 mg/day during the postoperative phase. Due to the thromboembolic risk associated with thalidomide, 81 mg aspirin was taken each morning on the same days as thalidomide if there were no contraindications. The protocol also contained dose modification recommendations for thalidomide toxicity. Patients with DVT were instructed to hold the drug until resolution to less than or equal to grade 1 toxicity, adequate therapeutic anticoagulation, and were then supposed to be restarted at a 50% dose reduction without further escalation.

**Figure 2:**
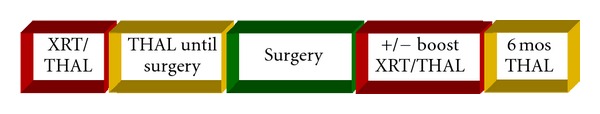
Treatment schema for RTOG 0330 cohort B. Neoadjuvant concurrent thalidomide (THAL) and radiation therapy (XRT) (stop THAL one week prior to surgery), followed by surgical resection, followed by adjuvant THAL for 6 months (post-op boost XRT if a positive margin).

Patients enrolled in cohort B were scheduled to receive neoadjuvant concurrent thalidomide and radiation followed by surgical resection and then adjuvant thalidomide for 6 months. Otherwise, thalidomide dosing, dose escalation, toxicity adjustments, and concomitant low-dose aspirin therapy were identical to cohort A. The treatment schema is summarized in Figure 2.

### 2.4. Surgery

The goal of surgical therapy was an oncologically complete resection, ideally with limb and function preservation, and negative margins, whenever achievable. Surgical resection of the primary tumor was to be performed 42–56 days after the last radiation dose. If the final pathology revealed positive soft tissue margins (not bone, nerve, or large blood vessels), surgical re-resection to obtain negative margins was strongly encouraged if it was not felt to have a major impact upon the patient's functionality. As it was felt that some extremity STS may require amputation simply to obtain grossly negative margins, primary amputation was not considered a local failure. However, amputation for a local recurrence after previous attempted limb preservation was scored as a local failure.

### 2.5. Statistical Analyses

The primary endpoint was treatment compliance. In 9514, 89% of patients received the protocol dose of radiation and 79% received all 3 preoperative cycles of MAID. For 0330, patients were considered compliant if they received at least 95% of the preoperative protocol dose of radiation, all 3 cycles of MAID (if applicable), and received thalidomide on 75% of the days during radiation as prescribed per protocol. A compliance rate of least 75% (for an individual cohort) was sufficient to consider a regimen for further study. For planning purposes, a 10% noncompliance rate was assumed with a noncompliance rate of 25% considered the highest acceptable rate. With 22 evaluable patients in each cohort, the plan had a type I error of 0.011 and type II error of 0.41.

In 9514, 78% of the 59 patients experienced a grade 4 hematologic toxicity and 3% had grade 5 toxicity (death). Twenty percent experienced grade 4 nonhematologic toxicities. In cohort A, no further increase in the rate of grade 4 hematologic toxicity was expected; an increase in the grade 4 nonhematologic toxicity rate to 35% would be considered unacceptable. The RTOG had not previously conducted a study in low-grade patients (cohort B). Therefore, the grade 4 nonhematologic toxicity rate was projected to be 10–15% with 25% considered unacceptable.

## 3. Results

### 3.1. Patient Characteristics

From June 17, 2004 until closure on March 21, 2007, 0330 accrued 23 primary STS patients from 10 different institutions. 16 patients were enrolled in cohort A and 7 patients in cohort B. One patient in cohort A was declared ineligible due to an unknown histologic grade. Patient and tumor characteristics for both cohorts are summarized in Table 1.

### 3.2. Neoadjuvant Chemoradiation and Surgical Resection

In cohort A, 93.3% of patients (14/15) received the planned 3 cycles of neoadjuvant MAID chemotherapy. The remaining patient was able to complete two cycles. All patients in both cohorts received 100% of the prescribed preoperative radiation dose (44 Gy for cohort A and 50 Gy for cohort B). In addition, all 22 patients underwent surgical resection of their primary STS. A pathologic complete response (no viable tumor) was seen in 20.0% of cohort A (3/15) and 28.6% of cohort B (2/7, both were a synovial sarcoma). On final pathology, positive resection margins were noted in 26.7% of cohort A (4/15) and 28.6% of cohort B (2/7). No patients underwent re-resection for a positive margin.

### 3.3. Neoadjuvant and Adjuvant Thalidomide

In cohort A, the median number of days on neoadjuvant thalidomide therapy was 30 (range 10–36), and the median cumulative dose was 6,000 mg (range 2,000–7,200). For cohort B, the median number of days on preoperative thalidomide therapy was 32 (range 3–36), and the median cumulative dose was 11,600 mg (range 600–19,400). Postoperatively, 60.0% of cohort A (9/15) received adjuvant thalidomide with a median cumulative dose of 25,300 mg (range 4200–73,000). Similarly, 57.1% of cohort B (4/7) received postoperative thalidomide with a median cumulative dose of 19,200 mg (range 17,100–36,600). All patients in both cohorts received 81 mg aspirin daily during thalidomide therapy.

### 3.4. Overall Treatment Compliance

In cohort A, 5/15 patients were considered non-compliant. In cohort B, 2/7 patients were considered noncompliant. Due to early study closure, this endpoint could not be fully evaluated per the protocol plan.

### 3.5. Treatment-Related Toxicity

Nonthromboembolic adverse events for both cohorts are summarized in Table 2. Excluding thromboembolic events, 27% of cohort A patients experienced a grade 4 nonhematologic adverse event, similar to the experience of RTOG 9514. Sixty-seven percent experienced grade 3 or 4 hematologic toxicity. There was no grade 5 toxicity. The only hematologic toxicity in cohort B was grade 1 in 28.6% (2/7).

None of the patients in cohort B experienced a thromboembolic event. In contrast, 40% of patients in cohort A (6/15) developed a thromboembolic complication. These events are summarized in Table 3. All thromboembolic events were in patients with a primary STS of the hip (*n* = 1) or lower extremity (*n* = 5). Two thirds of all events occurred in the preoperative setting: two bilateral pulmonary emboli (PE), one catheter-associated upper extremity deep venous thrombosis (DVT), and one incidental ipsilateral lower extremity DVT noted on preoperative restaging imaging. One of the patients who developed bilateral PE had not yet received any thalidomide therapy. Postoperatively, two patients developed an ipsilateral DVT within 30 days of surgery. There were no deaths from any of the thromboembolic events. Five of the patients were treated with anticoagulation and one of the preoperative patients underwent placement of an IVC filter.

Postoperative complications in cohort A included a grade 3 non-infectious wound complication in one patient, a grade 1 non-infectious wound complication in a second patient, and a grade 3 joint infection and grade 2 soft tissue necrosis of the lower extremity in a third patient. In Cohort B, 1 patient developed a grade 3 noninfectious wound complication and grade 3 blood infection, 1 patient developed a grade 1 noninfectious wound complication and grade 1 seroma, 1 patient developed a grade 2 non-infectious wound complication, and 1 patient developed a grade 3 seroma.

## 4. Discussion

Local tumor control with limb preservation for patients with high-risk STS has significantly increased through a combination of improved imaging, the multimodality approach of surgery and radiation therapy, and gradual improvements in these techniques. In contrast to local control, overall survival has not significantly changed over time. The risk for developing distant metastases with a high-grade STS is directly proportional to tumor size (34% for 5.1–10 cm, 43% for 10.1–15 cm, and 58% for 15.1–20 cm) [4]. RTOG 9514 was initiated in 1995 to examine the effects of intensive neoadjuvant MAID chemotherapy interdigitated with split courses of preoperative radiation followed by surgical resection and adjuvant systemic chemotherapy on local control and overall survival for high-risk STS. In this study, limb preservation was achieved in 92.2% of patients despite a median tumor size of 15 cm [1]. In addition, the pathologic complete response rate was an impressive 27%. Although there was no direct control group for comparison, the 71.2% estimated 5-year overall survival was much better than historical outcomes for comparable patients. Unfortunately, these results were not achieved without significant morbidity. Grade 4 hematologic toxicity was seen in 78% of patients and there were 3 treatment-related deaths. The significant, cumulative toxicity of this multimodality regimen was also evident by the fact that only 59% of patients completed all 6 planned cycles of MAID chemotherapy (3 preoperative and 3 postoperative) and 25% did not receive any postoperative chemotherapy.

In response to the results and limitations of 9514, the RTOG developed 0330. Thalidomide was chosen as a nontraditional therapeutic agent with antiangiogenic and potentially immunomodulatory properties, oral administration, and well-tolerated use in combination therapy for other malignancies such as multiple myeloma. Similar to 9514, cohort A (large, high-grade tumors) was developed to examine the effect of concurrent thalidomide on preoperative radiation therapy as well as whether or not long-term adjuvant thalidomide would be better tolerated than several cycles of postoperative MAID chemotherapy. RTOG 0330 also included large, low-grade tumors (cohort B) to evaluate similar endpoints, albeit using thalidomide and radiation without cytotoxic chemotherapy.

Prior to initiating 0330, there was concern regarding the association of thalidomide with a high incidence of venous thromboembolic events (VTE), such as deep venous thrombosis (DVT), and pulmonary embolus (PE), when used either alone or in combination therapy. A meta-analysis of 3,322 multiple myeloma patients treated with thalidomide alone, dexamethasone alone, combination therapy, or nonthalidomide/dexamethasone regimens showed that thalidomide was associated with a 2.6-fold increased risk for VTE while thalidomide with dexamethasone had an 8-fold increased risk [5]. Even more pertinent to 0330 was data by Zangari et al. showing that the incidence of DVT was 2.5% in multiple myeloma patients treated with a dexamethasone/thalidomide combination chemotherapy regimen that did not contain doxorubicin versus 16% percent in patients treated with the same regimen but including doxorubicin [6]. In the doxorubicin group, 35% of DVTs were associated with central venous access. There was also one nonfatal PE.

Due to the high incidence of VTE with thalidomide treatment for multiple myeloma, there has been interest in whether or not anticoagulation or antiplatelet therapy can reduce this risk. In a phase III study of newly diagnosed multiple myeloma patients randomized to induction doxorubicin containing chemotherapy with or without thalidomide, several cohorts were created based upon whether patients received anticoagulation prophylaxis with low-dose coumadin (1 mg per day) [7]. The rates of DVT were 14% for chemotherapy alone/no thalidomide or anticoagulation, 34% for chemotherapy with thalidomide/no anticoagulation, and 31% for chemotherapy with thalidomide/coumadin. The majority of DVTs occurred during the first cycle of treatment and coumadin did not reduce the risk (*P* = 0.7). Following treatment with systemic anticoagulation and resumption of chemotherapy, the DVT recurrence rate in the chemotherapy alone arm was 5% versus 11% in the thalidomide-containing group. A third cohort of chemotherapy alone versus chemotherapy/thalidomide with low-molecular-weight heparin (enoxaparin 40 mg subcutaneous daily) had equal rates of DVT at 15% (*P* = 0.81). The conclusion was that low-molecular-weight heparin, but not low-dose coumadin, was effective in reducing the risk of thalidomide-associated DVT and that thalidomide-containing therapy could be safely reinstituted following DVT treated with anticoagulation. Data supporting an antiplatelet approach includes a study from Baz et al. with an initial VTE incidence of 28.6% in multiple myeloma patients receiving pegylated doxorubicin, vincristine, dexamethasone, and thalidomide [8]. The protocol was then amended to include 81 mg aspirin daily, producing 3 cohorts: aspirin from the beginning of chemotherapy (group 1), aspirin at some point after starting chemotherapy (group 2), and no aspirin (group 3). The incidence of VTE was 19% in group 1, 15% in group 2, and 58% in group 3. As groups 1 and 2 were not statistically different, the hazard ratio for VTE with any aspirin use as compared to no aspirin was 0.22 (*P* < 0.001). There were also no bleeding complications associated with aspirin use.

Given the complexities of administering daily outpatient subcutaneous low-molecular-weight heparin over several weeks, a decision was made to use low-dose aspirin (81 mg) to reduce the VTE risk in 0330. However, the greatest challenge to deciding upon an appropriate VTE prophylaxis regimen for 0330 was the paucity of available data regarding expected baseline VTE rates during STS treatment. One retrospective review of children undergoing sarcoma treatment had a 11.5% VTE rate [9]. In a study of prophylactic IVC filter placement prior to adult musculoskeletal tumor surgery, the rates of DVT and PE were 11.8% and 0%, respectively, in a subset of 17 STS patients [10]. A review of VTE after orthopedic surgery in adult cancer patients (including non-STS surgery) noted a DVT rate of 14.2% and a PE rate of 0.6% that were comparable to these other studies [11]. In the sarcoma subgroup (bone and soft tissue), 15.6% of patients developed a DVT. One of the better assessments of sarcoma VTE risk is a retrospective study of 252 patients with a primary bone or STS (94 bone, 158 STS) [12]. The rate of DVT was 3.9%, PE was 1.2%, and fatal PE was 0.4%. Similar to our study, their VTE patients had a primary tumor located in the hip or lower extremity. Interestingly, 77% of the VTEs occurred prior to the definitive surgical resection of the sarcoma. In our study, 67% of cohort A VTEs also occurred preoperatively, including one DVT prior to even starting thalidomide therapy. As RTOG 0330 was based upon 9514, a reasonable comparison group for cohort A would also be the original 9514 patients. In 9514, there were only two grade 3 vascular adverse events (including one PE) for a VTE rate of 3.1% [1]. Although this would suggest that some of the VTE risk in 0330 came from the addition of thalidomide to the neoadjuvant therapy regimen, in retrospect, it may have been more prudent to administer daily low dose aspirin for VTE prophylaxis during all phases of the neoadjuvant therapy, not just the thalidomide.

## 5. Conclusion

So what was learned from 0330? Given the early closure of the study and the limited accrual to both cohorts, it is not possible to draw any conclusions regarding the effect of either treatment regimen on survival. Cohort B closed due to poor accrual, which was likely multifactorial, including the overall low incidence of STS as well as limited interest in adjuvant therapy trials for low-grade, lower-risk tumors. In cohort A, 40% of patients developed a VTE despite daily aspirin use during thalidomide treatment. Even excluding one event that occurred prior to initiating thalidomide and one catheter-related event, the rate of VTE was still 27%. As this rate is comparable to the high rates of VTE seen with combination chemotherapy/thalidomide for other cancers, daily low-dose aspirin did not seem to effectively reduce the VTE risk. VTE also limited the ability to deliver the prescribed adjuvant therapy (why 50% of cohort A patients did not receive adjuvant thalidomide). Although almost all of the cohort A patients were able to complete the planned treatment course of neoadjuvant MAID/thalidomide/radiation and there were no treatment related deaths, the pathologic complete response rate was only 20%. Consequently, thalidomide did not appear to increase the biologic effectiveness of the preoperative chemoradiation to justify the potential increased VTE risk. In contrast, none of the patients in cohort B experienced a VTE with thalidomide alone. Although patient numbers were very limited, the pathologic complete response rate to neoadjuvant therapy in cohort B was 29%, which is higher than one would expect for low-grade STS undergoing preoperative radiation alone. This raises a provocative question of whether thalidomide alone could function as a radiosensitizer in the treatment of some low-grade STSs.

## Figures and Tables

**Table 1:** Patient and tumor characteristics for cohort A and B patients in RTOG 0330.

	Cohort A (*n* = 15)	Cohort B (*n* = 7)
Median age (years)	49 (range 20–75)	47 (range 39–81)
Gender		
Male	8 (53.3%)	5 (71.4%)
Female	7 (46.7%)	2 (28.6%)
Median tumor size (cm)	12.8 (range 8.4–16.7)	10.0 (range 5.6–27.0)
Tumor site		
Upper extremity	1 (6.7%)	0 (0.0%)
Lower extremity	11 (73.3%)	5 (71.4%)
Buttock/hip	2 (13.3%)	1 (14.3%)
Abdominal wall	0 (0.0%)	1 (14.3%)
Back	1 (6.7%)	0 (0.0%)
Histology		
Liposarcoma	2 (13.3%)	3 (42.9%)
MFH	4 (26.7%)	0 (0.0%)
Synovial	2 (13.3%)	2 (28.6%)
MPNST	3 (20.0%)	0 (0.0%)
Other	4 (26.7%)	2 (28.6%)

MFH: malignant fibrous histiocytoma; MPNST: malignant peripheral nerve sheath tumor.

**Table 2:** Nonthromboembolic adverse events for cohort A and B patients in RTOG 0330.

	Cohort A (*n* = 15) Grade					Cohort B (*n* = 7) Grade				
Adverse event	1	2	3	4	5	1	2	3	4	5

Auditory/ear	0	1	0	0	0	0	0	0	0	0
Blood/bone marrow	2	1	5	5	0	2	0	0	0	0
Cardiac arrhythmia	1	2	1	0	0	0	1	0	0	0
Cardiac general	1	3	0	0	0	1	0	0	0	0
Coagulation	0	0	0	0	0	1	0	0	0	0
Constitutional symptoms	3	8	3	0	0	1	3	0	0	0
Dermatology/skin	2	7	2	0	0	1	3	1	0	0
Endocrine	1	0	0	0	0	0	0	0	0	0
Gastrointestinal	2	8	3	0	0	3	1	0	0	0
Hemorrhage/bleeding	1	1	0	0	0	1	1	0	0	0
Infection	0	2	2	0	0	0	1	2	0	0
Lymphatics	5	1	0	0	0	1	0	0	0	0
Metabolic/laboratory	3	5	2	0	0	1	1	0	0	0
Musculoskeletal/soft tissue	0	4	0	0	0	0	1	2	0	0
Neurology	2	4	3	0	0	0	4	0	0	0
Ocular/visual	3	0	0	0	0	0	1	0	0	0
Pain	2	8	0	0	0	1	1	0	0	0
Pending	1	0	1	1	0	0	0	0	0	0
Pulmonary/upper respiratory	0	2	0	0	0	0	0	0	0	0
Sexual/reproductive function	0	1	0	0	0	0	0	0	0	0

Worst nonhematologic	1	5	7	1	0	1	2	4	0	0
(%)	(6.7)	(33.3)	(46.7)	(6.7)	(0.0)	(14.3)	(28.6)	(57.1)	(0.0)	(0.0)

Worst overall	1	2	6	6	0	1	2	4	0	0
(%)	(6.7)	(13.3)	(40.0)	(40.0)	(0.0)	(14.3)	(28.6)	(57.1)	(0.0)	(0.0)

**Table 3:** Thromboembolic adverse events in 6 patients in cohort A of RTOG 0330.

Tumor site	Adverse event (AE)	AE grade	Timing	Comment
LE	Catheter-associated UE DVT	3	preop	prior to 3rd cycle MAID
Hip	Bilateral PE	4	preop	1st cycle MAID (no THAL)
LE	LE DVT	3	postop	4 weeks postop (ipsilateral)
LE	LE DVT	3	preop	presurgery MRI (ipsilateral)
LE	LE DVT	3	postop	1 week postop (ipsilateral)
LE	Bilateral PE	4	preop	2nd cycle MAID

LE: lower extremity; DVT: deep venous thrombosis; PE: pulmonary emboli; preop: preoperative; postop: postoperative; MAID: Mesna/Adriamycin/Ifosphamide/Dacarbazine; THAL: thalidomide; MRI: magnetic resonance imaging; UE: upper extremity.
